# MR diagnosis of vertebral artery dissection: value of 3D time-of-flight and true fast imaging with steady-state precession fusion imaging

**DOI:** 10.1007/s13244-012-0204-x

**Published:** 2012-12-02

**Authors:** Masafumi Kidoh, Takeshi Nakaura, Hiroki Takashima, Makoto Yoshikawa, Shouzaburou Uemura, Kazunori Harada, Toshinori Hirai, Yasuyuki Yamashita

**Affiliations:** 1 Diagnostic Radiology, Amakusa Medical Center, kameba 854-1, Amakusa, Kumamoto 863-0046 Japan; 2 Department of Diagnostic Radiology, Graduate School of Medical Sciences, Kumamoto University, Kumamoto, Japan; 3 Department of Neurosurgery, Amakusa Medical Center, Amakusa, Japan; 4 Department of Surgery, Amakusa Medical Center, Amakusa, Japan

**Keywords:** Fusion image, Vertebral artery dissection, MR angiography, True FISP, Magnetic resonance imaging

## Abstract

**Objectives:**

We hypothesized that 3D time-of-flight (TOF) and true fast imaging with steady-state precession (true-FISP) fusion imaging could provide more information regarding the arterial vessel wall. The purpose of this study was to compare the accuracy of lesion detection and the diagnostic confidence of VAD between TOF images alone and fused TOF and true-FISP images.

**Methods:**

Fifty patients were studied: 17 had VAD and 33 had vertebral artery hypoplasia. Fusion images of the vertebral artery were reconstructed using a workstation. A receiver-operating characteristic (ROC) analysis was conducted with a continuous rating scale from 1 to 100 to compare observer performance in VAD detection. Five radiologists participated in the observer performance test, and their performances with TOF images were compared with those using fused images.

**Result:**

The observers found that the mean areas under the best-fit ROC curve for TOF images alone and fused TOF images were 0.66 ± 0.05 and 0.93 ± 0.04, which were significantly different (*P* < 0.01).

**Conclusion:**

The fusion images provided more information regarding the arterial vessel wall. Fused images aided distinction between vertebral artery dissection versus vertebral artery hypoplasia.

***Key Points*:**

• *New MR techniques can help to differentiate flowing blood from static blood products*.

• *Fused TOF and true-FISP images differentiate the lumen and the arterial wall, improving diagnostic performance*.

• *Fused images may be superior to time-of-flight MR angiography alone*.

## Introduction

Vertebral artery dissection is a potential cause of posterior circulation ischemia that requires high-spatial-resolution imaging for the definitive diagnosis [1]. Digital subtraction angiography (DSA) remains the gold standard for assessment of the vertebrobasilar arteries, with excellent spatial and temporal resolution [2, 3]. However, risks associated with conventional angiography include vascular injury, intracerebral complications, contrast medium nephrotoxicity and exposure to ionizing radiation. Therefore, non-invasive diagnostic techniques such as CT angiography (CTA) and magnetic resonance imaging (MRI) with MR angiography (MRA) are typically used. CTA has been shown to have high sensitivity and specificity for the diagnosis of vertebral artery dissection [4]. However, the accuracy of CT in the evaluation of acute ischaemic lesions in the posterior cranial fossa remains limited [5].

Three-dimensional (3D) time-of-flight (TOF) MRA is important as a non-invasive examination to diagnose blood flow in the brain. TOF MRA mainly reflects the blood flow within the artery, representing the inner contour of the artery. Intramural dissecting haematoma shows a typical evolution of signal intensity related to the paramagnetic effects of the products of hemoglobin breakdown and is frequently isointense to surrounding structures [6–8]. Therefore, the diagnostic performance of TOF in the detection of vertebral artery dissection is poor.

True fast MR imaging with steady-state precession (true-FISP) is a coherent steady-state technique that uses a fully balanced gradient waveform to recycle transverse magnetization. True FISP is extremely rapid relative to black-blood T1WI and T2*WI and fat-suppressed T1WI. True FISP offers a high signal-to-noise ratio and imaging efficiency; true FISP provides reliable examination of blood vessels without the need for intravenous administration of contrast medium [9]. However, true FISP is not suitable for the imaging of fast flow velocity because the reduction of signal intensity on true FISP is remarkable with a flow velocity of 50–100 cm/s, which corresponds to arterial flow velocity [10]. Therefore, true FISP is not suitable for visualizing the inner contour of the artery but can provide information regarding the outer surface appearance of the intracranial arterial wall.

There is still no consensus regarding the optimal method for diagnosing the dissection of intracranial arteries with MRI [11]. Previous reports suggested that the criteria of increase in the external diameter of the artery and narrowing of the lumen offer high diagnostic accuracy [12, 13]. Additionally, previous reports have suggested the usefulness of fused images to depict the different anatomical information at one view [14–16]. Therefore, the combination of true-FISP and TOF MRA may be useful for more accurate diagnosis in patients with conditions such as vertebral artery hypoplasia or vertebral artery dissection. We hypothesized that the TOF and true-FISP fusion imaging could provide more information regarding the arterial vessel wall.

The purpose of this study was to compare the accuracy of lesion detection and the diagnostic confidence of vertebral artery dissection between TOF images alone and fused TOF and true-FISP images.

## Methods

### Patients

This prospective study was performed with institutional review board approval. Details of the study were explained, and written informed consent was obtained from all patients before the MRI examinations, including possible anonymous use of data for research purposes.

Clinical MRI examinations were performed between April 2009 and April 2012. Our study included 50 patients, 30 males and 20 females, ranging in age from 41 to 88 years (mean 68.1 years) (Table 1). Seventeen patients with vertebral artery dissection were included in the study. Thirty-three consecutive asymptomatic patients who were diagnosed with vertebral artery hypoplasia from 1 July to 17 September 2010 were also assigned to a control group.

**Table 1:** Patient characteristics

	Vertebral artery dissection	Vertebral artery hypoplasia
Number of patients	17	33
Male:female ratio	12:5	18:15
Age (years)	69.7 ± 12.6	65.7 ± 13.2

### Diagnosis

The final diagnosis of vertebral artery dissection was based on (1) compatible clinical signs and symptoms of vertebral artery dissection with definite angiographic, CTA or MRA findings of dissection in the vertebral artery; (2) no evidence of luminal irregularities, stenosis or occlusions in vertebral arteries or the other cervical and intracranial arteries that would be suggestive of atherosclerosis.

Compatible clinical signs and symptoms of vertebral artery dissection included sudden-onset occipital headache, neck pain or brain stem ischaemic symptoms [17]. The imaging findings for diagnosing vertebral artery dissection were as follows [18]: (1) a narrowed centric or eccentric lumen surrounded by crescent-shaped, mural thickening and an associated increase in external diameter; (2) an abrupt or tapered occlusive lumen and an associated increase in external diameter; (3) an aneurysmally dilated lumen or an alternatively dilated and narrowed lumen with or without crescent-shaped mural thickening or an intimal flap; and (4) healing or progression of the initial lumen abnormality on follow-up. Increased external diameter was evaluated by comparing it with the segment proximal to the dissection.

In addition to MRI examinations, conventional angiography and CTA were also performed in some patients (Table 2).

**Table 2:** Diagnostic modalities used for the diagnosis of vertebral artery dissection

	Number of patients
Conventional angiography	1/17
CTA	5/17
Contrast-enhanced MRI	7/17
Follow-up non-contrast-enhanced MRI	17/17

Thirty-three patients with vertebral artery hypoplasia who were asymptomatic were reviewed as a control group. Vertebral artery hypoplasia was defined as a lumen diameter of ≤2 mm [19].

### Imaging

We obtained 3D TOF images and true-FISP images using 1.5-T MRI (Magnetom Avanto; Siemens Medical Systems, Germany). The imaging parameters were as follows: 3D TOF: 18/2.84 (TR/TE); flip angle, 20°; section thickness, 0.77 mm; readout bandwidth, 300 Hz/pixel; rectangular field of view, 210 mm; matrix, 256 × 256; unenhanced true FISP: 6.82/3.41 (TR/TE); flip angle, 70°; section thickness, 0.7 mm; readout bandwidth, 501 Hz/pixel; rectangular field of view, 210 mm; matrix, 256 × 256. We reconstructed fused 3D TOF and true-FISP images of the vertebral artery for qualitative image analysis using a 3D-imaging workstation (Synapse Vincent, Fuji Film Medicals, Japan). Fusion images were presented using a linear rainbow color scale with seven colors ranging from purple to red. Images were displayed and saved as bitmaps.

### Study design

Five radiologists participated in the observer performance test, and their performances with TOF images were compared with their performances using TOF and fused TOF and true-FISP images. The observers were three board-certified radiologists with 9–19 years of experience (mean, 12.7 years) and two radiology residents with 5–6 years of experience with brain MRI (mean, 5.5 years). All observers read brain MRI images regularly. Radiologists were blinded to the patients’ identities, clinical histories and MR sequence parameters. The reading time was not limited. The observers first analysed only TOF images and next analysed two separate sets of TOF and fused TOF and true-FISP images. The images were presented in random order in each case. Radiologists were asked whether the image showed vertebral artery hypoplasia or vertebral artery dissection, and then they marked their confidence level regarding the likelihood of vertebral artery dissection by using a continuous rating scale [20]. They were asked to try to use the rating scale consistently and uniformly. Specific criteria for vertebral artery hypoplasia or vertebral artery dissection were a large mismatch between the region of the TOF and the region of the true FISP because previous reports suggested that the criteria of increase in the external diameter of the artery and narrowing of the lumen offer high diagnostic accuracy [12, 13]. All cases were reviewed in a random order on an LCD monitor with a spatial resolution of 1,600 × 1,200 (RadiForce R22, Nanao) using our PACS (Synapse, Fuji Film Medicals, Japan).

### Quantitative image analysis

We calculated the differences between lumen signal (TOF) and vessel size (true FISP). Non-tortuous portions of the hypoplastic vertebral artery were measured on transverse images. Measurement was also performed at the most enlarged portion of the dissected artery. At each portion, three independent measurements were averaged. We calculated the differences between TOF and true FISP of vertebral artery hypoplasia and vertebral artery dissection using the following formula: vessel size - lumen signal.

### Statistical analysis

The decision confidence ratings were analysed using ROC techniques. The data were analysed using DBM-MRMC, version 2.2, software (C.E. Metz, The University of Chicago, Chicago, IL, USA) [21–27]. To find significant differences among the methods and readers (multireader-multimodality ROC analysis), the jackknife method was applied. To compare the differences between TOF and true FISP among the two protocols, we used two-tailed Student’s *t*-test. A difference with *P* < 0.05 was considered significant.

## Results

**Fig. 1:**
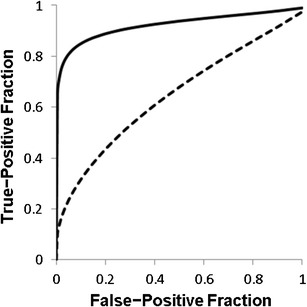
Mean ROC curves for all observers who detected vertebral artery dissection. Mean area under the ROC curve increased from 0.66 ± 0.05 (TOF images, *dashed line*) to 0.93 ± 0.04 (fused images, *solid line*); this difference was statistically significant (*P* < 0.01)

There was no clinically problematic misregistration between TOF and true-FISP fusion images. For the five observers, the mean area under the best-fit ROC curve (Az) values for TOF images alone and TOF and fused TOF and true-FISP images were 0.66 ± 0.05 (SD) and 0.93 ± 0.04, respectively (Fig. 1 and Table 3). The difference was significant (*P* < 0.01).

**Table 3:** Area under receiver-operating characteristic curve values for diagnosis of vertebral artery dissection on TOF and fused images

Observer no.	TOF images	Fused images
	AUC	AUC
1	0.73	0.99
2	0.75	0.95
3	0.61	0.78
4	0.64	0.97
5	0.58	0.93
Mean (SD)	0.66 (0.05)	0.93(0.04)

The differences between TOF (lumen signal) and true FISP (vessel size) of vertebral artery dissection were significantly larger than those of the hypoplastic side of vertebral artery (*P* < 0.01) (Table 4).

**Table 4:** The differences between TOF and true FISP

	Vertebral artery hypoplasia	Vertebral artery dissection	*P* value
The differences between TOF and true FISP (mm)	0.3 ± 0.2	3.6 ± 1.6	< 0.01

**Fig. 2:**
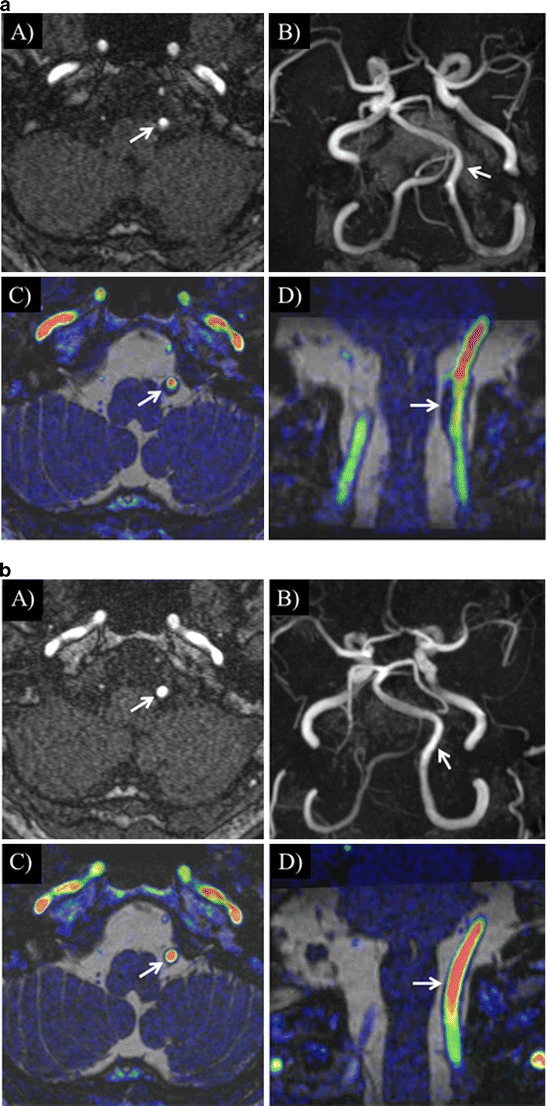
**a** A 63-year-old man complaining of headache. The left vertebral artery dissection on 3D TOF (*A* and *B*) is not clear (*arrow*). Its shape is not equal to what is evident on fused images. Fused image shows enlargement of the overall vessel diameter with an intramural haematoma in the left vertebral artery. Fused image enabled precise assessment of the relationship between the inner and outer contours of the vessels (*arrow*). (*A*) 3D TOF, (*B*) 3D TOF (MIP), (*C*) TOF and true-FISP fusion (axial), (*D*) TOF and true-FISP fusion (oblique). **b** Four months later, he underwent follow-up MRI. Vertebral artery dissection was confirmed on imaging follow-up by monitoring normalization of the vessel lumen. Follow-up MRI scan indicated normalization of the vessel lumen (*arrow*). (*A*) 3D TOF, (*B*) 3D TOF (MIP), (*C*) TOF and true-FISP fusion (axial), (*D*) TOF and true-FISP fusion (oblique)

**Fig. 3:**
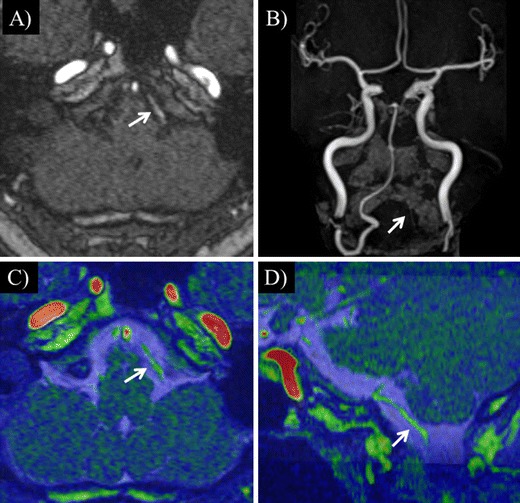
A 67-year-old man. A case of left hypoplastic vertebral artery. The left vertebral artery is not seen clearly on 3D TOF (**a** and **b**) (*arrow*). A small and hypoplastic left vertebral artery is confirmed on fused imaging (**c** and **d**). Fused image shows no abnormalities along the course of the vertebral artery (arrow). **a** 3D TOF, **b** 3D TOF (MIP), **c** TOF and true-FISP fusion (axial), **d** TOF and true-FISP fusion (oblique)

Representative cases with vertebral CIS artery dissection and vertebral artery hypoplasia are shown in Figs. 2 and 3.

In addition to the TOF-MRA and true-FISP imaging, T1-weighted, T2-weighted, T2-star-weighted, fluid-attenuated inversion recovery and diffusion-weighted imaging were performed in all 50 patients. Ten patients with vertebral artery dissection underwent MR imaging with the additional sequences (Table 5).

**Table 5:** Additional sequences in the imaging of vertebral artery dissection

Patient no.	Dynamic 3D-VIBE	CE-T1WI	FS-CE-T1WI	CE-TOF-MRA	3D TSE (SPACE)
1	〇	〇			
2	〇				
3	〇			〇	
4	〇			〇	〇
5	〇	〇		〇	
6		〇	〇		〇
7		〇	〇		
8		〇		〇	
9		〇		〇	〇
10					〇

*VIBE* volumetric interpolated breath-hold examination, *CE* contrast-enhanced, *FS* fat saturated, *TSE* turbo spin echo, *SPACE* sampling perfection with application optimized contrasts using different flip angle evolution

## Discussion

Fused images enable clear visualization of the vertebral artery, and the relationship of the inner and outer contours of vessels can be assessed precisely. Fused images aided distinction between vertebral artery dissection versus vertebral artery hypoplasia. Fused images may have a supplementary role to TOF MRA images in the diagnosis of vertebral artery dissection.

Quantitative image analysis revealed that the differences between lumen signal and vessel size of vertebral artery dissection were significantly larger than those of hypoplastic side of vertebral artery. Dissections of the vertebral arteries are caused by a primary intramural haematoma or by penetration of blood into the arterial wall through a primary intimal tear [28, 29]. Blood usually enters the media at the site of intimal injury, and the dissection usually extends cranially in the same direction as the bloodstream [30–32]. The intramural haematoma usually compresses the true lumen of the artery and causes enlargement of the external diameter of the artery.

There is no consensus regarding the optimal method for diagnosing the dissection of vertebral arteries. The diagnosis of dissection at the site of vertebral artery lesions is based on the clinical history and the findings of conventional angiography, CT or MRI. On T2-weighted MRI, evidence of intra-arterial lesions, such as mural haematoma, intimal flap and plaque, are characteristic findings of vertebral artery dissection. On three-dimensional contrast-enhanced images, the double lumen at the site of dissection is visualized [33]. On T1-weighted and fluid-attenuated inversion recovery MRI images, intraluminal vertebral artery haematomas can be detected, and the pearl-and-string and the double lumen signs are characteristic MRA findings of vertebral artery dissection [33–36].

The diagnostic performance of TOF in the detection of vertebral artery dissection is poor. TOF MRA is currently the most commonly used pulse sequence in the MR evaluation of intracranial arteries. However, the sensitivity of TOF MRA for the detection of vertebral artery dissection is 60 % [12, 37]. In comparison, multidetector 3D-CTA has good accuracy for the diagnosis of vertebral artery dissection, with sensitivity and specificity of 100 % and 98 %, respectively. If the vessel dissection enlarges distally, the MR signals in the pseudo-lumen of dissected vessels are affected by the turbulence of blood flow, and TOF MRA may not clearly depict blood flow within the pseudo-lumen [38–40]. Thus, TOF MRA may underestimate the area of dissection.

True FISP is not suitable for visualizing the inner contour of the artery but is suitable for visualizing its outer contour. The true-FISP technique was originally designed for improved visualization of the cerebrospinal fluid [41]. Attributes of the true-FISP sequence include a relatively high image signal-to-noise ratio and rapid data acquisition, which yields diminished sensitivity to motion. These attributes are favorable for depicting the outer surface appearance of the arterial wall. However, pulsation artifacts (inhomogeneous signal) within the arteries are frequent on true-FISP images [42]. Additionally, the signal intensity on true-FISP is reduced when the flow velocity is more than 30 cm/s [10]. Therefore, true FISP is not suitable for the imaging of fast flow velocity; that is, true-FISP MR imaging alone is not suitable for visualizing the inner contour of the artery. True FISP reflects the outer surface appearance of the intracranial arterial wall, whereas TOF MRA mainly reflects the blood flow within the artery, representing the inner contour of the artery.

Use of fused imaging is a good diagnostic method for vertebrobasilar disease. Three-dimensional TOF MRA evaluation of the absence or decrease in the blood flow signal may not clearly discriminate vertebral artery hypoplasia from vertebral artery dissections. Therefore, we consider that evaluation of the dissection of vessels should include examination of both the inner and the outer contours of the vessels. The fused imaging reflects the outer surface appearance of the intracranial arterial wall and the inner contour of the artery, whereas TOF MRA mainly reflects the blood flow within the artery, representing the inner contour of the artery. This may be the difference from the established vascular imaging methods such as DSA, TOF MRA, CTA or black blood MRA [43–45], which disclose the vascular inner contour. The relationship between the inner and outer contours of the vessels is clearly depicted on fused images. Therefore, we think this imaging technique provides additional information in the detection of vertebral artery dissection.

Our study had limitations. The major limitations to this pilot study include the small sample and lack of a normal vessel group. To confirm the coherence between TOF and true FISP, we might have to consider a control group of normal vessels. Second, complete blinding of the readers to the sequence type was not possible because their signal characteristics are quite typical. Third, TOF imaging may be affected by flow changes compared with contrast-enhanced MRA. Therefore, we cannot exclude the possibility that luminal stenosis was overestimated on fused images. Lastly, fusion imaging of TOF and true FISP only evaluates the relationship between the inner and outer contours of vessels, without differentiating between arterial wall thickening due to arteriosclerosis and dissected arteries. To exclude atherosclerotic diseases, clinical presentations, supportive radiologic evidence and follow-up imaging are needed.

In conclusion, the fusion images provided more information regarding the arterial vessel wall. Fused images aided distinction between vertebral artery dissection versus vertebral artery hypoplasia. Fused images may have a supplementary role to play in TOF MRA images in the diagnosis of vertebral artery dissection.
